# Early Breast Cancer Risk Assessment: Integrating Histopathology with Artificial Intelligence

**DOI:** 10.3390/cancers16111981

**Published:** 2024-05-23

**Authors:** Mariia Ivanova, Carlo Pescia, Dario Trapani, Konstantinos Venetis, Chiara Frascarelli, Eltjona Mane, Giulia Cursano, Elham Sajjadi, Cristian Scatena, Bruna Cerbelli, Giulia d’Amati, Francesca Maria Porta, Elena Guerini-Rocco, Carmen Criscitiello, Giuseppe Curigliano, Nicola Fusco

**Affiliations:** 1 Division of Pathology, European Institute of Oncology IRCCS, 20141 Milan, Italy; mariia.ivanova@ieo.it (M.I.); carlo.pescia@unimi.it (C.P.); konstantinos.venetis@ieo.it (K.V.); chiara.frascarelli@ieo.it (C.F.); eltjona.mane@ieo.it (E.M.); giulia.cursano@ieo.it (G.C.); elham.sajjadi@ieo.it (E.S.); francescamaria.porta@ieo.it (F.M.P.); elena.guerinirocco@ieo.it (E.G.-R.); 2 Division of New Drugs and Early Drug Development for Innovative Therapies, European Institute of Oncology IRCCS, 20141 Milan, Italy; dario.trapani@ieo.it (D.T.); carmen.criscitiello@ieo.it (C.C.); giuseppe.curigliano@ieo.it (G.C.); 3 Department of Oncology and Hemato-Oncology, University of Milan, 20122 Milan, Italy; 4 Department of Translational Research and New Technologies in Medicine and Surgery, University of Pisa, 56126 Pisa, Italy; cristian.scatena@unipi.it; 5 Department of Medical-Surgical Sciences and Biotechnologies, Sapienza University of Rome, 00185 Rome, Italy; bruna.cerbelli@uniroma1.it; 6 Department of Radiological, Oncological and Pathological Sciences, Sapienza University of Rome, 00185 Rome, Italy; giulia.damati@uniroma1.it

**Keywords:** breast cancer, early breast cancer, risk stratification, pathology, biomarkers, artificial intelligence, predictive algorithms, deep learning

## Abstract

**Simple Summary:**

Risk assessment in early breast cancer is critical for clinical decisions, but defining risk categories poses a significant challenge. The integration of conventional histopathology and biomarkers with artificial intelligence (AI) techniques, including machine learning and deep learning, has the potential to offer more precise information. AI applications extend beyond detection to histological subtyping, grading, and molecular feature identification. The successful integration of AI into clinical practice requires collaboration between histopathologists, molecular pathologists, computational pathologists, and oncologists to optimize patient outcomes.

**Abstract:**

Effective risk assessment in early breast cancer is essential for informed clinical decision-making, yet consensus on defining risk categories remains challenging. This paper explores evolving approaches in risk stratification, encompassing histopathological, immunohistochemical, and molecular biomarkers alongside cutting-edge artificial intelligence (AI) techniques. Leveraging machine learning, deep learning, and convolutional neural networks, AI is reshaping predictive algorithms for recurrence risk, thereby revolutionizing diagnostic accuracy and treatment planning. Beyond detection, AI applications extend to histological subtyping, grading, lymph node assessment, and molecular feature identification, fostering personalized therapy decisions. With rising cancer rates, it is crucial to implement AI to accelerate breakthroughs in clinical practice, benefiting both patients and healthcare providers. However, it is important to recognize that while AI offers powerful automation and analysis tools, it lacks the nuanced understanding, clinical context, and ethical considerations inherent to human pathologists in patient care. Hence, the successful integration of AI into clinical practice demands collaborative efforts between medical experts and computational pathologists to optimize patient outcomes.

## 1. Introduction

Early breast cancer (EBC) refers to organ-confined tumors with the limited involvement of axillary lymph nodes, aligning with the TNM stages T1-3, N0-2, and M0, as defined by the American Joint Committee on Cancer (AJCC) [1,2]. The precise risk definition of EBC is a challenging but crucial task for driving clinical decision-making [3]. Despite significant efforts in clinical trials such as monarchE (NCT03155997) and NATALEE (NCT03701334), and dedicated working groups such as IRIDE to identify high-risk EBC and guide treatment, there are currently no widely employed guidelines [4,5] or specific recommendations for the definition of high- and low-risk EBC in clinical practice, using definitions that are capable of estimating patients’ prognosis with high sensitivity and specificity. Common parameters that are used for risk stratification include histopathological factors (e.g., histological subtype, grade, tumor size, number of metastatic lymph nodes, presence of lymph–vascular invasion (LVI), and tumor-infiltrating lymphocytes (TILs)), hormone receptors (HRs), HER2, Ki67 status, BRCA1/2, and gene expression profiling data [6,7,8,9]. Many currently used genomic signatures have high positive predictive values for defining low-risk groups but have less reliable negative predictive values [10]. Consequently, these signatures are generally imperfect for decisions about intensifying treatment, although they are more dependable for identifying lower-risk populations [11]. Broader “omics” approaches and circulating biomarkers are gradually being integrated within the currently validated information for EBC risk stratification [3,12,13]. In such an evolving scenario, artificial intelligence (AI) technologies, including machine learning (ML), deep learning (DL), and convolutional neural networks (CNNs), emerge as transformative tools to enhance predictive algorithms precisely assessing the risk of recurrence in EBC [14,15,16]. These AI applications, particularly in digital and computational pathology, allow for the extraction of subvisual morphometric phenotypes, potentially leading to diagnostic breakthroughs [17,18,19,20,21,22,23]. However, their application both in clinical studies and real-world clinical practice faces several unresolved challenges [24]. This article aims to explore the intersection of traditional pathology and AI in the definition of novel and more reproducible algorithms for EBC risk profiling.

## 2. Prognostic and Predictive Models on Digitalized H&E-Stained Tissue Slides

AI in pathology diagnosis has been defined as a part of “clinical decision support systems” and has shown its efficacy in pathology reports’ improvement, aiding in tumor classification and grading [25,26,27]. In recent developments, scientists have engineered ML and DL algorithms for the identification and categorization of breast cancers [28,29].

### 2.1. Breast Cancer Detection and Quantification

Several commercially available AI platforms are designed for breast core biopsy histological assessment [30,31,32]. Among these, the GALEN algorithm can analyze entire core needle biopsy whole slide images (WSIs) and detect various types of breast lesions, including invasive/in situ carcinoma, its non-obligate precursors, such as atypical hyperplasia, and benign findings like sclerosing adenosis, fibroadenoma, and fat necrosis [31,32,33,34]. Developed through an ensemble of CNNs trained on over 2 million labeled image patches from manual annotations on 2153 hematoxylin and eosin (H&E)-stained slides, the algorithm demonstrated robust performance with an impressive area under the curve (AUC) of 0.99 for detecting invasive carcinoma (specificity and sensitivity of 93.6% and 95.5%, respectively) and an AUC of 0.98 for detecting ductal carcinoma in situ, reporting the effective clinical validation of a multi-feature AI algorithm aiding pathologists in precisely identifying both invasive and in situ breast carcinoma [30,32]. The implementation of AI-based tools for detecting women with non-aggressive ductal carcinoma in situ (DCIS) could be employed to avoid surgery and/or radiotherapy without jeopardizing the positive outcomes for women with high-risk DCIS who require treatment [33]. A key advantage of implementing clinical decision support systems is their reliance on H&E slides solely, which potentially accelerates the diagnostic process, reduces the expense associated with costly genetic testing, and prevents both over- and under-treatment [29,31,32,33].

### 2.2. Histological Classifier

A structured DL-based multi-classification method has recently been proposed to autonomously assess breast cancer histological subtypes, including not only ductal carcinoma and lobular carcinoma but also other subtypes, such as rare mucinous carcinoma and papillary carcinoma [28]. The model underwent validation on the BreaKHis dataset that includes 7909 images and eight sub-classes of breast cancers [35], demonstrating a commendable performance level with an average accuracy of 93.2% [28,30]. These types of models should be refined using a training approach that involves transfer learning from natural images, a standard method employed in DL analysis [34,36]. Generally, ensemble learning and embedded fusion CNN models outperform alternative integration methods, making them a powerful tool for accurate feature extraction and histopathology-based image classification [36,37]. Incorporating an online mutual knowledge transfer strategy as a fusion approach within CNNs holds promise for enhancing different types of breast cancer detection [36]. Multi-classification models are likely to relieve pathologists’ workload, providing their longitudinal validation in dedicated clinical studies [34,38].

### 2.3. Cancer Grading

For the grading of breast cancer (BC), various solutions have been proposed, such as DL-based models to identify and count mitoses [39] and a CNN model for BC grading based on nuclear features [40] and tubule formation, exhibiting correlation with histologic grade and recurrence risk in early-stage estrogen receptor-positive (ER+) BC, as determined by a molecular test (Oncotype DX) [41]. Precise ML models for mitosis detection are rapidly evolving, refining cell-level annotations to streamline the annotation process for WSIs [42]. In a new first large-scale study, three different mitosis scoring methods have been evaluated with the help of AI in a clinical setting including two separate large BC cohorts [43]. Six certified pathologists have been equipped with an online annotation tool, performing the annotations for the diverse phases and morphologies of mitotic figures, as well as identifying atypical mitotic figures, excluding potential mimickers (e.g., lymphocytes, stromal cells, artifacts), which resulted in an annotation of a total of 7916 mitotic figures [43]. Statistically significant correlations of a mitotic activity index (MAI) were observed between pathologists and an automated MAI (r = 0.8, *p* < 0.001) and demonstrated this scoring method as a standalone predictor of survival [43]. Additionally, the AI-scored MAI has demonstrated a strong correlation with the Ki67 proliferation index [43]. Importantly, different AI-based mitosis scoring methods for predicting outcomes in adjuvant chemotherapy (CT) have shown strong associations with outcomes in CT-naïve patients [20]. However, among patients receiving adjuvant CT, only the MAI maintained a significant association with outcomes (HR 2.35, 95% CI 1.88–2.93; *p* < 0.001), while other scoring methods lost their significance [43].

### 2.4. Sentinel Lymph Node

Other instruments have been developed to facilitate BC staging, specifically in the assessment of sentinel lymph node H&E slides [20]. CAMELYON16 [44] and CAMELYON17 [45] datasets were used for DL-based lymph node metastases detection in BC and have demonstrated superior performance compared to pathologists in a competitive challenge [46]. They excel in identifying small tumor lesions, showcasing the effectiveness of the DL-based methodology [45]. The method was further enhanced by combining a patch-level CNN-based metastasis detector and slide-level lymph node classifier, achieving a quadratic weighted kappa score of 0.9203, suggesting high concordance [47]. One significant limitation of the studied AI systems is their focus on detecting a single primary pathological lesion or metastatic cell. Consequently, these systems may fail to identify other rare yet relevant histological features, leading to a potential increase in false positive results when using additional AI-based digital tools. For instance, hypertrophic lymphoid follicles, reactive venules or capillaries, and macrophages may be erroneously classified as micrometastases [15]. Moreover, non-histological elements like paraffin debris, bubbles, or stains may also be misinterpreted as metastatic lesions by the algorithm [15]. To mitigate these errors, incorporating an experienced pathologist’s supervision is fundamental [48]. Indeed, the successful implementation of these tools in routine practice upon validation could significantly speed up pathology report delivery, carrying numerous benefits for the patient as diagnosis-aiding tools [30].

### 2.5. Tumor-Infiltrating Lymphocytes (TILs)

TILs carry an important prognostic value in BC, with more robust data in human epidermal growth factor receptor 2-positive (HER2+) and triple-negative BC (TNBC) [49]. Several AI algorithms have been proposed for facilitating TIL scoring based on WSIs, including a deep learning CNN-based method, classifying cell nuclei to distinguish lymphocytes [50,51]. In contrast, the role of TILs in ER+HER2- breast cancers remains elusive [52] and may identify tumors exhibiting unfavorable clinicopathological factors, such as high tumor grade, more advanced stage, and younger age, resulting in poorer clinical outcomes. A supervised deep learning model analysis of H&E WSIs has recently been tested on 2231 ER+HER2- early-stage BC patients [53]. The results revealed TILs as an independent predictor of worse outcomes in these patients using a multivariate Cox regression analysis and the reliability of AI in TIL assessment [53]. Similarly, an ML-based cluster analysis of immune cell subtypes in BC has shown distinct immune responses to tumor growth, suggesting the algorithm’s potential for disease management and survival prognostication [54]. In one of the largest notable studies focused on DL, a computational stain for TIL identification has been developed, encompassing TIL patterns from 4759 subjects in The Cancer Genome Atlas (TCGA) across 13 different cancer types [55]. The computationally stained TILs exhibited a correlation with both pathologist visual assessments and molecular estimates [55]. Furthermore, the study revealed that TIL patterns were associated with tumor and immune molecular characteristics, cancer type, and overall outcome [55]. However, AI-based algorithms may face similar challenges to those faced by pathologists during visual assessment. These challenges include the identification of TILs in nontumor or “in situ” tumor areas, the presence of necrosis, and the variability of preanalytical workflows. Additionally, these challenges are further complicated by technical factors, such as the lack of standardized parameters for data acquisition. [56]. AI-assisted TIL quantification remains a promising tool when handled by an experienced pathologist; however, forecasting clinical outcomes based on pre-treatment histopathologic images remains a challenging endeavor, hindered by the incomplete comprehension of the tumor immune microenvironment [52,56,57].

### 2.6. BRCA and Homologous Recombination Deficiency

BCs harboring homologous recombination deficiency (HRD) display a phenotype characterized by the failure of a specific DNA repair pathway, resulting in high genomic instability [58]. In the context of BC, HRD is frequently associated with BRCA1 and BRCA2 alterations [59]. HRDs resulting from mutations in those genes are known as predictive markers for the response to PARP inhibitors (PARPis) [60,61]. Although most sporadic and hereditary BRCA1 cancers carry the TNBC phenotype (ER−, PR−, HER2−), the majority of hereditary BRCA2 cancers are of a luminal type (HR+, HER2-) [62,63]. Considering the population of BC potentially affected, as well as the logistical and economic challenges of large-scale genomic screening for HRD, a robust DL approach for HRD prediction using digitized HE-stained tumor slides was recently developed [64]. The authors proposed a DL image-based approach, implementing H&E-stained WSIs from a large series of TNBC and luminal-type BC with a genomically defined homologous recombination status [64]. The authors succeeded in demonstrating the capability of the algorithm to predict HRD with high accuracy with an AUC of 0.86, additionally identifying morphological HRD-associated features, opening avenues to new phenotypic hypotheses [64]. For example, HRDs for TNBC appear to be recapitulated by a high content of TILs and necrosis, while retraction figures correlated with proficient homologous recombination. Of note, DL has also been applied to WSIs to detect BRCA mutations in high-grade ovarian cancer, based on a tumor segmentation method [65]. The authors suggest that relevant information for the prediction of BRCA mutations lies more in the tumor context, rather than cell morphology, and that the developed DL tool could be used for prescreening [65]. An emerging study has led to the development of an interpretable BC molecular subtype classification framework, based on DL utilizing multi-omics datasets (moBRCA-net) [66]. The authors integrated a complex of three omics datasets: gene expression, DNA methylation, and microRNA expression data, considering their biological relationships. A self-attention module was applied to each omics dataset to capture the relative importance of features, further transforming them based on their learned importance, enabling moBRCA-net to predict the BC subtype [66]. Their results demonstrated that the proposed algorithm exhibited significantly improved performance compared to other methods, underscoring the effectiveness of multi-omics integration [66]. A concept of “Earlier than Early” BC detection has been recently presented in Israel, where the authors aimed to identify BRCA mutation carriers by applying AI-based analysis to consecutive MRI scans [67]. The model successfully classified 65% of the cancerous foci, primarily TNBC [67]. If validated, this approach could enable an “earlier than early” BC diagnosis in BRCA pathogenic variant carriers [67]. Overall, patients’ prognosis and treatment responses are largely influenced by the pre-treated tumor ecosystem, and machine learning can integrate its multi-omics landscape into predictive models [68]. Additional combinations of convolutional networks may lead to an increase in datasets’ robustness [69].

## 3. Prognostic and Predictive Models on Immunohistochemistry-Stained Tissue Slides

Immunohistochemistry (IHC) assays play a key role in categorizing, guiding decisions, and predicting outcomes in BC patients [3,70]. However, the performance of IHC is resource-intensive, time-consuming, costly, contingent on specific tissue-handling protocols, and relies on pathologists’ subjective interpretation [71,72]. To address the latter concern, digital image analysis (DIA) has been widely employed in interpreting IHC staining [57,71,72]. While image analysis through ML is increasingly utilized across various pathology applications, it has yet to be suggested as a replacement for chemical-based assays in molecular detection [71,72]. Ongoing research endeavors seek to integrate both molecular and morphological tumor characteristics, aiming to enhance the prognostic and predictive capabilities of ML methodologies [57].

### 3.1. Hormone Receptors

The evaluation of hormone receptor (HR) status is both a prognostic and predictive factor in BC and a crucial step in tailoring therapy in BC patients [73,74,75]. The implication of DIA in ER and PgR receptors’ evaluation by IHC image analysis is plausible after the massive success of Ki67 automated scoring, given that all these markers exhibit nuclear expression [76,77]. A recent study has demonstrated increased interobserver agreement among pathologists in IHC HR status assessment when using AI support [76]. The largest AI-implementing study to date included 10 participant pathologists from eight sites, six WSI scanners/microscopes, and three staining systems [76]. A major advantage of the study was not requiring manual fine-tuning for the provided image by the pathologist, using the same configuration across all tissue images. The pathologists agreed with the proposed AI assistance results in 93.2% of ER/PgR cases, indicating the potential of relying on automated cell counting with AI assistance after manual regions of interest (ROI) definition [76]. The study examined the potential and accuracy of AI tools, both with and without human intervention, while also exploring their limitations. The findings demonstrated the safety of utilizing the assistance tool, with statistical significance [76]. Similar results have been demonstrated earlier for ER and PgR scoring using IHC-stained images engaging a deep neural network composed of an encoder, a decoder, and a scoring layer [78]. The authors stated the excellent performance of the created network, potentially facilitating the human error-prone and time-consuming process and aiding in screening and diagnosis in the early detection of BC [78]. However, caution should be taken in the case of faint staining or a new emerging sub-class of ER-low cases [79], where AI still has the margin of generating false negative results [80]. Therefore, emerging studies aim to evaluate the potential for predicting the molecular expression of biomarkers in cancer tissues by solely relying on the tissue architecture observed in digitized specimens stained with H&E [72]. One of the biggest analyses of this kind included the publicly available database of 20,600 digitized H&E-stained sections of 5356 patients with BC [72]. The authors developed an ML technique, named morphological-based molecular profiling (MBMP), employing logistic regression to investigate the associations between tissue morphology and biomarker expression, while subsequently utilizing a deep CNN for predicting biomarker expression in analyzed tissues [72]. In this study, MBMP demonstrated comparable predictive efficacy to IHC for at least half of the patients, and the findings indicated that the accuracy of predictions is likely to be enhanced with the expansion of training datasets [72]. Similarly, a multiple-instance learning-based deep neural network was used to develop the algorithm for BC HR status determination [81]. The study involved H&E WSIs from 3474 patients, achieving an impressive AUC of 0.92, with a positive and negative predictive value of 0.932 and 0.741, respectively, for both sensitivity and specificity [81]. The deployment of authorized IHC scoring/predicting AI algorithms could offer a swift, accurate, and cost-effective method for the simultaneous profiling of multiple biomarkers in cancer. Integrating these algorithms into a digital pathology laboratory information system would further enable a streamlined and automated workflow [72,82].

### 3.2. Ki67

One of the first and most widely used AI algorithms in pathologists’ practice was Ki67 proliferation index scoring, provided by many freely available platforms [76,77,83]. The scoring of Ki67, a cell proliferation marker, is of the utmost predictive and prognostic importance in BC [84,85]. No “gold standard” has been defined for Ki67 index determination [83,86]. Visual assessment is inherently subjective and susceptible to various factors, including individual experience [83]. The widely employed visual “eyeballing” method is time-efficient, and numerous studies have underscored substantial interobserver variability associated with this approach [87,88]. The utilization of DIA tools has the potential to facilitate a faster and more standardized evaluation of Ki67 [86,87]. Several freeware image analysis instruments for nuclear staining algorithms implemented in WSI analysis are available for free, with no additional equipment needed, and show excellent agreement with the manual count [71,83,86,89]. The improved agreement among pathologists in assessing Ki67 in breast tumors has also been reported when employing digital AI tools, as opposed to the subjective naked-eye assessment, especially in heterogeneous marker expression patterns [83,87,90]. Presently suggested approaches primarily involve the computation of Ki67 using a deep learning model, which establishes interpretations for detecting hotspots. The identified ROI is subsequently employed to segment relevant cells through conventional image processing methods [91]. An improved AI performance has been observed in a study of 329 tissue microarray tumor cores from different BC subtypes [92]. The excellent reproducibility by correlation with manual pathologists’ scoring could be achieved due to sequential Ki67 and cytokeratin IHC staining. This approach permitted precise tumor cell recognition, by superimposing cytokeratin-highlighted epithelium [92]. The method is promising in stratifying high-risk BC patients, if approved, and would cut the costs of genomic-based prognostic assays. However, the major downside is that the proposed algorithm might not be suitable for less common BC types (i.e., metaplastic) [92]. Overall, it is anticipated that the Ki67 comparison standard will become the benchmark method for the routine interpretation of immunohistochemical Ki67 results in breast cancer in the near future [83,86].

### 3.3. HER2

An accurate evaluation of human epidermal growth factor receptor 2 (HER2) expression is crucial for effective breast cancer treatment [93,94,95,96]. The current approach is transitioning from a binary HER2 assessment to recognizing HER2-low tumors (1+ or 2+ with negative in situ hybridization (ISH)), as emphasized by data with novel treatment, specifically trastuzumab deruxtecan in HER2-low BC [97]. This shift has fundamentally transformed the treatment approach for HER2-low disease, with the proven effectiveness of antibody–drug conjugates (ADCs) serving as a significant addition to the therapeutic arsenal [98,99]. The focus now turns to the importance of precise HER2 evaluation, which can be facilitated by advanced AI technologies [27,57,100]. Various methods are employed in HER2 IHC AI algorithms: some of them are based on tumor cells’ segmentation, while others evaluate HER2 membrane staining intensity and patterns [101,102,103]. These AI approaches have been utilized in studies to differentiate between HER2-positive and HER2-negative cases [101,102,103]. The Visiopharm DIA algorithm assessed 612 digitized HER2 invasive BC specimens demonstrating 87.3% concordance with pathologists [103]. The authors emphasize that HER2 IHC DIA demonstrated a capability to accurately discriminate between HER2 fluorescent ISH (FISH)-positive and -negative cases, suggesting that the HER2 copy number may be more important in predicting HER2 protein expression and the response to anti-HER2-targeted therapy [103]. Following this, numerous AI models have been created with the sole purpose of predicting the HER2 status through the analysis of H&E-stained slides [81,104,105], with some of them even being able to predict trastuzumab response in BC at an accuracy that may benefit clinical evaluations [106]. The latest research data reinforce the argument for the automated quantification of mutation-specific protein overexpression in H&E-stained digital pathology and underscore the significance of employing multi-stage machine learning pipelines to enhance both robustness and interpretability in the analysis [104,106]. One of the most recent studies has introduced a spatial transformer network (STN) for weakly localizing critical image features, subsequently utilizing a vision transformer-based deep learning architecture, to detect HER2 expression without IHC staining [107]. The authors attempted to evaluate the HER2 staining in BC based on H&E images only and reported success in HER2 expression staging (AUC 0.9202 ± 0.01, precision 0.922 ± 0.01, sensitivity 0.876 ± 0.01, and specificity 0.959 ± 0.02 over five-fold cross-validation with a 95% confidence interval (CI)) [107]. As stated, this approach significantly outperformed conventional vision transformer models and state-of-the-art models (*p* < 0.001) [107]. Nevertheless, the primary limitations of the previous studies stem from algorithms adhering to a canonic dichotomous HER2 expression classification (positive/negative). Detecting HER2-low cases on WSIs poses a greater challenge [30]. Certain attempts in smaller cohorts have achieved success, demonstrating algorithms’ ability to score both IHC and in situ hybridization slides [101,108,109]. However, the most significant disparities between AI and pathologist evaluations still lie within a score range of 0–1+ [108]. Recent investigations in this direction suggest the feasibility of overcoming this as well. A recent analysis of 246 HER2 IHC BC slides in two rounds (without and with the help of AI assistance) has shown the consistency of pathologist-reviewed results and AI results [110]. Surprisingly, AI showed superior results in the precision for HER2 0 (0.93) and HER2 1+ scoring (0.93) detection [110], mainly due to a more clear separation of the HER2 ultra-low subgroup (score 0 with incomplete and faint staining in ≤10% of tumor cells) [100]. More success has been achieved with the Paige algorithm, which was first validated for the detection of prostate cancer WSIs [111]. Recent studies are now implementing this algorithm in BC H&E-stained WSIs, demonstrating that it can effectively differentiate between breast cancers lacking both the HER2 protein and mRNA (HER2-null) and tumors with low levels of HER2 expression [112]. The Guideline From the College of American Pathologists comments on the opportunities to use quantitative image analysis for diagnostic testing, emphasizing the urge for the validation of the emerging algorithms with all performance, interpretation, and reporting steps being supervised by an expert pathologist [113].

**Figure 1:**
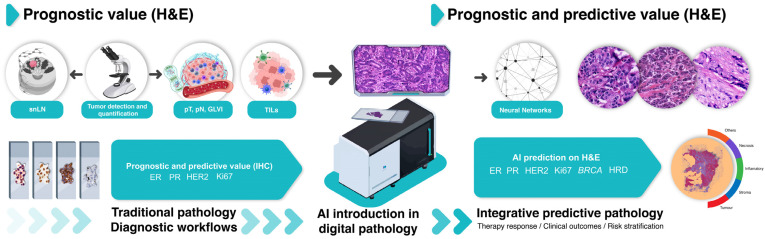
Established and evolving AI-developed approaches of EBC risk definition, involving various biomarkers’ analysis and clinicopathologic features. Traditional histopathological characteristics, detected on H&E staining, form the core of the pathology report, providing essential prognostic information: tumor identification and quantification, tumor size, lymph node involvement, histological grade (according to the Nottingham system), lymphovascular invasion, and sentinel lymph node status. Some pathologists may report TILs, although current recommendations do not suggest basing therapeutic strategies on this biomarker IHC assessment of the hormone receptor and HER2 status (according to ASCO/CAP guidelines), and the Ki67 proliferation index is essential to assign BCs to the luminal/non-luminal molecular classification and to guide treatment choices with both prognostic and predictive implications. In cases suggestive of hereditary BC syndrome, HRD and BRCA1/2 testing is recommended. The developing landscape of AI-based DL algorithms involves the creation of neural networks, capable of predicting IHC status on H&E slides without an actual IHC staining, followed by genomic status and therapy response prediction, risk assessment, and improved patient prognostic stratification. Abbreviations: AI, artificial intelligence; BC, breast cancer; EBC, early breast cancer; DL, deep learning; H&E, hematoxylin and eosin; IHC, immunohistochemistry; pT, primary tumor size; pN, regional lymph node involvement; G, histological grade; LVI, lymphovascular invasion; snLN, sentinel lymph node; TILs, tumor-infiltrating lymphocytes; ER, estrogen receptor; PR, progesterone receptor; HRD, homologous recombination deficiency.

An overview of available prognostic and predictive models in digital pathology applied on BC WSIs is represented in Table 1, and a schematic overview of the established and evolving AI-developed approaches of EBC risk definition, involving various biomarkers’ analysis and clinicopathologic features, is represented in Figure 1.

## 4. Integration of Multiple Predictive Tools into Diagnostic Neural Networks

AI has shown its potential in prognosis and therapeutic response prediction based on the histological features of the tumor by linking images directly to it [46]. The assessment of the architectural organization and spatial configuration of various tissue types through graphical approaches has sparked significant interest in predicting clinical outcomes [114].

### 4.1. Predicting Therapy Response

Revisiting the context of pathology, a DL-based H&E image analyzer, Lunit SCOPE, has been developed for identifying and quantifying various histologic components from H&E-stained WSIs [117]. The authors hypothesized that the cell proportions analyzed by the DL algorithm could serve as a potential prognostic and predictive biomarker for adjuvant CT in early-stage HR-positive breast cancer. Notably, in patients deemed high-risk by Lunit SCOPE, adjuvant CT demonstrated a significant prolongation of disease-free survival (HR 0.35, 95% CI 0.15–0.86, *p* = 0.0161) and overall survival (HR 0.22, 95% CI 0.05–0.95, *p* = 0.0254) [117]. This suggests that the risk score provided could be a significant predictive biomarker for the effectiveness of adjuvant CT [117]. One of the newest studies introduced an image-based H&E-only prognostic marker for early-stage luminal/HER2-negative BC, termed “BRACE” [118]. The marker was derived from AI-based assessments of heterogeneity in BC at a detailed level, utilizing the capabilities of deep learning [118]. The BRACE marker effectively stratified patients for both distant metastasis-free survival (*p*  =  0.001, C-index: 0.73) and BC-specific survival (*p*  <  0.0001, C-index: 0.84), with a prediction accuracy comparable to established indices (the Nottingham Prognostic Index and Magee score) [119]. Given the results, the authors suggest the potential of the BRACE marker in identifying luminal BC patients likely to benefit from adjuvant CT [118]. Of note, while ER positivity by IHC traditionally guides endocrine therapy selection in BC, it may not consistently correlate directly with activated ER signaling activity, which is a more accurate predictor of therapy responsiveness [120]. Hence, one of the most recent studies sought to predict the BC endocrine treatment response from H&E staining based on estrogen receptor 1 (ESR1) signaling activity [121]. The study analyzed 1082 BC samples from the TCGA Pan-Cancer dataset and determined ER signaling activity using available RNA sequencing (RNA-seq) data [121]. Later, a DL model was trained using processed H&E-stained images and ER signaling activity scores and was applied to predict ER activity in breast cancer patients. Higher predicted ER activity scores in ER+/HER2- patients correlated with longer progression-free survival. The trained models robustly predicted prognosis without the need for RNA-seq or microarray data analyses, potentially reducing diagnostic workflow costs [121]. Recently, a novel machine learning model of HR-positive BC recurrence risk was developed based on the immune microenvironment analysis of data in 2338 HR+HER2- BC cases from publicly available datasets [122]. As a result, a nine-gene signature has been established to stratify high-risk tumors with an association to poor endocrine and CT response [122]. Nevertheless, patients’ stratification according to molecular marker expression is a promising research line; some authors claimed that a single-gene markers approach (as HER2 expression) is insufficient [123]. After developing 16 machine learning algorithms and eight molecular profiles resulting in 128 models’ creation, they proposed the one with the best performance—CART (classification and regression tree), which combined four selected miRNA isoforms in a non-linear manner, predicting BC patients’ response to doxorubicin [123]. The predictive efficacy of the model was pinpointed by comparison to HER2 expression, which was found less predictive [123]. On the other hand, an attempt to apply an ML model to predict the pathologic complete response (pCR) to neoadjuvant therapy in HER2+ BC patients based on a subset of clinical features only has demonstrated that clinical features alone are inadequate for defining a useful support system in clinical pathways [124].

### 4.2. Risk Assessment: Genomics and Beyond

The combination of multiple tools with DL capabilities is gaining popularity among researchers, representing a promising multidisciplinary approach with broad implications. The latest study combining automated BC detection with an AI-based analysis of 11 markers using multiplex fluorescence IHC (mfIHC) in 1404 invasive breast cancers of no special type allowed for a swift and reliable analysis of multiple prognostic parameters [123]. The automated breast cancer detection framework accurately distinguished normal and malignant glands with 98.4% accuracy, identifying five biomarkers (PR, ER, androgen receptor (AR), GATA3, PD-L1) to be associated with prolonged overall survival (*p* ≤ 0.0095 each) showing strong prognostic relevance (*p* < 0.0001) and being an independent risk factor in multivariate analysis (*p* = 0.0034) [123]. These data suggest that automated BC detection in combination with an AI-based analysis of mfIHC could provide a rapid and reliable analysis of multiple prognostic parameters [123]. Predicting clinical outcomes presents a complex challenge, with a limited feasibility of utilizing pre-treatment histopathologic imaging [57]. In a recent study involving HER2+ BC and TNBC patients, a combination of H&E and multiplex IHC images (PD-L1, CD8+, and CD163+) was examined using automated feature extraction [120]. Features extracted from the tumor immune microenvironment and clinical data were employed to train machine learning models for the precise prediction of the response to neoadjuvant CT (AUC for HER2+ patients = 0.8975; AUC for TNBC patients = 0.7674), demonstrating superior algorithm performance compared to pathologists [120]. Recent research is worth mentioning that aimed to analyze the receptor status in primary breast cancer and matched brain metastases (BM), establishing radiomic signatures to predict the receptor status of the latter [121]. The authors have conducted the machine learning-based radiomic signature implementation using contrast-enhanced MRI brain images, to predict the BM receptor (ER, PR) and HER2 status. Radiomic signatures have demonstrated a potential for noninvasively predicting the BM status with high accuracy, sensitivity, and specificity [121]. These data are especially exciting in light of the poor prognosis of BC patients with BM, frequent receptor discordance between primary tumor and BM, and absence of screening strategies for these patients [121,122]. Another set of opportunities involves genome-wide association studies aiming to identify genetic mutations influencing specific traits. Recently, a new approach utilized AI to investigate the link between germline genomic mutations and breast cancer risk, establishing the Damage Assessment of Genomic Mutations (DAGM) framework [123]. The DAGM model calculates cumulative effects on gene expression and generates Activity Profiles of Signaling Pathways (APSP) scores, indicating the impact on cellular pathways and assessing breast cancer risk. Although the model relies on publicly available data and lacks extensive real-world validation, ongoing AI model development holds promise for providing convenient and accurate assistance in predicting cancer risks [123]. It is emphasized that these analyses necessitate scalable algorithms for large patient cohorts and addressing latent confounders, to achieve optimization tools from deep learning [124].

## 5. Pitfalls and Prospectives

The latest guidelines from the European Society for Medical Oncology (ESMO) underscore the importance of patient follow-up in in EBC management [125]. Nevertheless, the absence of data from recent randomized trials using modern imaging suggests that surveillance protocols should consider patient needs, costs, and healthcare system burdens [125]. Additionally, recent advancements in DIA are found to be efficient and time-saving for pathological diagnosis, demonstrating good agreement, particularly in cases of intratumor heterogeneity [71,86,89]. The main issues in the integration of AI into the diagnostic pathology process are related to the user dependence of the algorithms with a need for external control [25,64]. Most AI diagnostics rely on operator proficiency, with the quality of input data and the necessity for external expert control being the primary determinants. Identifying order sets and the algorithmic rules within AI has proven to be notably challenging [57,126]. Unexperienced users may develop workarounds that compromise data which leads to jeopardizing data integrity [25,127]. Tissue sample size could also represent a potential issue (i.e., core biopsies), as well as the subjectivity of “hotspot” selection for training the algorithm [86,127]. The presence of artifacts stemming from sampling, slide preparation, and slide digitalization can impede computational analysis and lead to erroneous data interpretation. Consequently, there is considerable interest in the development of artifact detection tools, exemplified by the open source HistoQC tool [128]. HistoQC integrates image metrics, edge detection, and other classifiers (e.g., pen detection) to discern artifact-free regions on digitized slides, exhibiting 94–97% concordance with expert pathologists. This implementation serves to enforce quality control in the selection of WSIs suitable for computational analysis. The presence of artifacts notably impacts machine learning (ML) methodologies, which currently lack a level of nuanced understanding akin to the human interpretation of slides. This deficiency arises from factors including personal expertise and the incorporation of supplementary data and considerations. Furthermore, given the heterogeneity of breast cancer cells, it has been reported that AI may not comprehensively identify each tumor cell, leading to potential cell misclassification [15,83,127]. This emphasizes the ongoing significance of human expertise and intervention in ensuring the accuracy and effectiveness of AI-driven diagnostic processes. The successful implementation of AI tools into clinical practice requires a multidisciplinary approach due to the complexity of challenges faced [129]. The discrepancy between developmental and operational datasets may result in a lack of generalizability, highlighting the importance of appropriate data application and continuous updates [130,131,132,133]. Financial considerations, including initial setup costs and ongoing expenses, continuous training for new personnel, and regular system updates to align with the latest advancements in knowledge, pose additional challenges to healthcare institutions [18,25,134]. Moreover, optimizing informatics and technical support systems is essential to maximize the performance of AI algorithms, thereby maximizing the quality and efficiency of diagnoses [25,134].

## 6. Conclusions

In addressing AI-based technologies in EBC risk stratification, it is crucial to emphasize pathologists’ trust in utilizing such tools effectively. This trust hinges on tailored education and training, pivotal for widespread clinical adoption. However, despite offering robust tools for automation and analysis, AI technologies still lack the nuanced understanding, clinical context, and legal and ethical considerations that pathologists bring to patient care [20,57,135,136,137,138]. Training AI models with manual annotations, for example, is indeed crucial to refine algorithm performances, yet it is a complex and time-consuming endeavor, among other challenges. Moreover, while AI tends to simplify data visualization into more discrete forms, especially in image analysis, the final interpretation remains multifaceted. Thus, building trust among pathologists toward AI requires not only technical proficiency but also a comprehensive understanding of its limitations and the indispensable role of human insight in clinical decision-making. Therefore, a collaborative approach that combines AI automation with human expertise is ideal for optimizing patient care in oncology. Ultimately, leveraging AI advancements can expedite breakthroughs in cancer treatment, benefiting both patients and healthcare providers [20,57].

## Figures and Tables

**Table 1:** An overview of the available prognostic and predictive models in digital pathology applied on BC WSIs.

WSI Source	Classifier	Features Assessed	Test Dataset (Number of Cases)	Validation Dataset (Number of Cases)	Reference
H&E	Galen breast	Histologic subtype, grade, DCIS, ADH, TILs	Maccabi (1090)	Institute Curie/Maccabi (171/270)	[31]
H&E	CSDCNN	Malignant vs. benign	BreaKHis (21)	BreaKHis (61)	[28]
H&E	MuDeRN	Malignant vs. benign	BreaKHis (81)	BreaKHis (81)	[34]
H&E	DeepMitosis	Mitotic count	MITOSIS 2012/2014 (50/960)	MITOSIS 2014 (240)	[39]
H&E	CNN *	Histological grade	TCGA/CHTN (397/1537)	METABRIC (1807)	[40]
H&E	DL classifier *	Histological grade	Original with ER+ samples (174)	Original with ER+ samples (11)	[41]
H&E	ML classifier *	Mitotic count	Nottingham ER+ HER-/TCGA (1715/757)	Nottingham ER+ HER2- (859)	[43]
H&E	CAMELYON16	SLN metastasis	Original (270)	Original (129)	[44]
H&E	CAMELYON17	SLN metastasis	Original (899)	Original (100)	[114]
H&E	NRK-ABMIL	SLN metastasis	CAMELYON16/17 (129/500)	CAMELYON16/17 (129/500)	[45]
H&E	DL classifier *	TILs	Cleveland Clinic Foundation (120)	Cleveland Clinic Foundation (14)	[51]
H&E	DL classifier *	TILs	Nottingham ER+/HER2- (2231)	University Hospital Coventry and Warwickshire (318)	[53]
H&E	CIBERSORT	TILs	METABRIC/TCGA (1903/1075)	Original (204)	[54]
H&E	CNN *	TILs	TCGA (5455, 13 cancer types)	TCGA (5455, 13 cancer types)	[55]
H&E	DL classifier *	HRD	Institute Curie/TCGA (715/673)	Institute Curie/TCGA (715/673)	[64]
H&E	CNN *	HER2	Original (26)	Original/TCGA (26/45)	[104]
H&E	HEROHE	HER2	Original (150)	Original (150)	[105]
H&E	CNN *	HER2	Publicly available datasethttps://bupt-ai-cz.github.io/BCI (4873)	Publicly available datasethttps://bupt-ai-cz.github.io/BCI (4873)	[107]
H&E	Metafer 4 classifier	HER2 amplification	Original (CHU-Hôpital du Saint-Sacrement) (96)	Original (CHU-Hôpital du Saint-Sacrement) (64)	[109]
H&E	AI assistance too l *	HER2-low	Original (Pathologie Institut Enge AG) (97)	n/a	[108]
H&E	AI assistance tool *	HER2-low	Original (Fourth Hospital of Hebei Medical University) (246)	n/a	[110]
H&E	ReceptorNet	ER, HER2	Australian Breast Cancer Tissue Bank/TCGA (2535/1014)	Australian Breast Cancer Tissue Bank/TCGA (2728)	[81]
H&E	MBMP CNN	ER, PgR, HER2	Genetic Pathology Evaluation Centre TMA (20,600)	Genetic Pathology Evaluation Centre TMA (20600)	[72]
H&E	CNN *	HER2, response to trastuzumab	Original/TCGA (188/668)	TCGA (569)	[106]
H&E	Paige	HER2	Original (Memorial Sloan Kettering Cancer Center) (1479)	Memorial Sloan Kettering Cancer Center (1479)	[112]
IHC	QuPath	Ki67	Original (660, 280, 41)	Original (660, 280, 41)	[71,89,115]
IHC	AI assistance tool *	Ki67	Original (Hebei Medical University) (150)	Original (Hebei Medical University) (150)	[83]
IHC	AI assistance tool *	Ki67	Original (72)	Original (72)	[77]
IHC	NuclearQuant	Ki67	Original (Bellvitge University Hospital) (136)	Original (Bellvitge University Hospital) (136)	[87]
IHC	CNN *	Ki67	Original, (Lower Silesian Oncology Center) (95)	Original, (Lower Silesian Oncology Center) (95)	[91]
IHC	KiQuant	Ki67	Original (Vall d’Hebron University Hospital) (329)	Original (Vall d’Hebron University Hospital) (329)	[92]
IHC	Automated ER DIA	ER	Original (97)	Original (97)	[80]
IHC	HscoreNet	ER, PgR	Original (Tata Medical Center) (600)	Original (Tata Medical Center) (600)	[78]
IHC	AI assistance tool *	Ki67, ER, PgR	Original (Institute of Hematopathology Hamburg) (204)	n/a	[76]
IHC	Sunnyoptic ARM50	HER2	Original (Hebei Medical University) (1022)	Original (Hebei Medical University) (1022)	[116]
IHC	ImmunoMembrane	HER2	Original (Helsinki University Central Hospital) (750)	Original (Helsinki University Central Hospital) (750)	[101]
IHC	HER2-CONNECT	HER2	Original (University of Copenhagen, Ohio State University Wexner Medical Center) (462, 612)	Original (University of Copenhagen, Ohio State University Wexner Medical Center) (462, 612)	[102,103]

WSI, whole slide image; H&E, hematoxylin and eosin; IHC, immunohistochemistry; DCIS, ductal carcinoma in situ; ADH, atypical duct hyperplasia; TILs, tumor-infiltrating lymphocytes; CNN; convolutional neural network; TCGA, The Cancer Genome Atlas; CHTN, Cooperative Human Tissue Network; DL, deep learning; ML, machine learning; SLN, sentinel lymph node status; HRD, homologous recombination deficiency; AI, artificial intelligence; HER2, human epidermal growth factor receptor; ER, estrogen receptor; PgR, progesterone receptor; 2; TMA, tissue microarray; DIA, digital image analysis; *, generic names.

## Data Availability

No new data were created in this study. Data sharing is not applicable to this article.
